# Minutes of the Ohio State Dental Society

**Published:** 1886-12

**Authors:** 


### MINUTES.

### Minutes of the Ohio State Dental Society.

SECOND ANNUAL MEETING OF THE OHIO STATE DENTAL SOCIETY, TOLEDO, OHIO, OCTOBER 26, 1886.

Society called to order by President, at 10.30 A. M. Prayer was offered by Rev. Dr. W. W. Williams, of Toledo. Minutes of last session read and approved.

Dr. H. A. Smith moved that visiting members of the profession from other States be granted the privilege of the floor. Motion carried.

On account of non-arrival of members of Committee on Membership, the President appointed the following committee to act temporarily:

DR. H. A. SMITH, DR. A. F. EMMINGER,
DR. H. H. HARRISON.

On motion of Dr. Sillito, the Society adjourned to meet at 2 P. M.

OCTOBER 26, 1886.

AFTERNOON SESSION.

Society called to order by President, at 2:15 P. M. Minutes of last session read and approved.

The following persons having made application for membership, and being recommended by Board of Directors, were severally elected to membership in this Society:

Drs. S. D. Potterf, C. R. Taft, E. H. Rafensperger, J. A. Lupton, J. F. Siddall, Jas. I. Taylor.

Dr. H. A. Smith then moved that Dr. P. G. C. Hunt, of Indianapolis, Ind., and Dr. J. S. Cassidy, of Covington, Ky., be elected to honorary membership. Motion carried.

The first subject, viz.: "What Should be the Treatment in Cases of Impacted Third Molars?" was then called.

Dr. C. R. Butler read a paper on the above subject.

The subject was discussed by Drs. Keely, Harrison, and others.

The second subject was then called.

On motion of Dr. J. Taft, the paper of Dr. W. J. Younger, of San Francisco, was read by Dr. Metcalf.

The paper was discussed by Drs. Harroun, Taft, Smith, and others.

Adjourned to 7:30 P. M.

EVENING SESSION.

Society called to order at 8 P. M., Vice-President Harrison in the chair. Minutes of last session read and approved.

The following named persons having been recommended by the Board of Directors, were severally elected to active membership in this Society:

Drs. J. A. Stipp, Geo. H. Wilson, Geo. Dennis, E. G. Wage, T. C. Leiter, J. W. Lyder, C. M. Wright, S. Clipinger.

On motion of Dr. C. H. Harroun, the following persons were elected to honorary membership:

Dr. A. P. Metcalf, of Kalamazoo, Mich., and Dr. J. A. Robinson, of Jackson, Mich.

Discussion on Dr. Younger's paper was then resumed by Drs. J. A. Robinson, Harroun, Keely, and others.

Adjourned to meet at 9 A. M.

OCTOBER 27, 1886.

MORNING SESSION.

Society called to order at 9.30 A. M., Vice-President Harrison presiding.

Minutes of last session read and approved. Upon the recommendation of Board of Directors, the following named persons were elected to active membership:

A. S. Condit, E. D. Schebel, Ira Brown, R. L. Evans, F. S. Whitsler, Jas. A. Stockton, J. R. Bell, Wm. A. Sedgwic, W. H. Whitsler, J. F. Dennis.

The following persons were elected to honorary membership:

Dr. J. A. Watling, Michigan; C. B. Porter, Michigan; T. W. Brophy, Chicago; W. W. Allport, Chicago; S. B. Brown, Fort Wayne, Ind.

Dr. F. H. Rehwinkle then gave a clinic demonstrating the Herbst method of filling teeth.

Adjourned to meet at 2 P. M.

AFTERNOON SESSION.

Reading of minutes dispensed with. Dr. Brophy, of Chicago, demonstrated his method of filling approximal cavities, using his improved matrix.

On motion the Society proceeded to the election of officers for the ensuing year, with the following result:

President, H. H. Harrison, Cadiz.
First Vice-President, C. M. Wright, Cincinnati.
Second Vice-President, Jere. E. Robinson, Cleveland.
Treasurer, Geo. W. Keely, Oxford.
Secretary, J. R. Callahan, Hillsboro.

MEMBERS OF BOARD OF DIRECTORS.

Drs. E. G. Betty, F. C. Runyan, Chas. Welch, W. H. Hague.

Moved by Dr. Keely that the Secretary be authorized to appoint an Assistant Recording Secretary to perform such duties as the Secretary may assign him. Motion Carried.

Drs. J. Taft and F. H. Rehwinkle were re-elected members of the Ohio State Board of Dental Examiners.

Upon ballot it was decided to hold the next meeting of this Society in Springfield, Ohio.

Adjourned to meet at 8 P. M. 27th.

OCTOBER 27, 1886.

EVENING SESSION.

Society called to order at 8:15, Vice-President Harrison in the chair. Minutes of the two previous sessions read and approved.

Dr. Allport, of Chicago, then read a paper discussing the merits of the Herbst method of filling teeth.

Upon invitation, Dr. Brophy, of Chicago discussed the application of the matrix.

On motion the subject was passed.

On motion of Dr. H. A. Smith, the second subject was called. No papers appeared.

Moved that the fifth subject be reopened, Carried.

Upon invitation, Dr. Harlan, of Chicago, expressed his views on the fifth subject. Subject further discussed by Drs. Dorrance, Land, Keely, and others.

Adjourned to meet at 9 A. M.

OCTOBER 28, 1886.

MORNING SESSION.

Society called to order at 9:25 A. M. by President. Minutes of last stated meeting read and approved.

On motion the officers elect were installed.

On motion, Dr. J. A. Robinson read a paper entitled, Operative Dentistry. Paper was discussed by Drs. Butler, Harroun, and others.

Dr. C. R Butler then read a paper entitled, "Items of Interest."

The third subject was then taken up and discussed by Drs. Harlan, Smith, Taft. On Motion, third subject was passed.

The seventh subject was then taken up.

Dr. Talbot, of Chicago, demonstrated his method of correcting irregularities of the teeth.

Dr. Keely presented a number of models of irregularities, and demonstrated his method of correcting the same.

The following resolutions were presented and adopted:

*Resolved,* That a vote of thanks be tendered Mr. Groff, proprietor of Boody House, for courtises shown the members of the Society, and for the free use of committee rooms.

*Resolved*, Further, that a copy of these resolutions be delivered to Mr. Groff.

*Resolved*, That a vote of thanks be tendered the Honerable City Council of Toledo, and to the City Clerk, for the courtesies they have shown the Ohio State Dental Society in giving us the use of the City Council Chamber and the Committee Rooms adjoining, and

*Resolved*, Further, that the resolutions be spread upon the minutes, and a copy thereof be transmitted to the City Clerk.

The State Examining Board made the following report:

Five gentlemen presented themselves before the Board for examination, viz.:

C. C. Scott, Celina; S. G. Downing, Lorain; E. E. Dayball, Salem; C. H. Griffin, Ravenna; Stephen Hate, Parkman.

These gentlemen were all examined orally and in writing. Messrs Scott, Downing, Dayball, and Griffin passed satisfactorily, while action on Mr. Hate was laid over for one year.

Fees to the amount of $15.00 were collected.

Disbursments: Stationery, postage, etc., $2.15, leaving a balance of $12.85, which was turned over to the Treasurer of the Society.

E. G. Betty, Secretary.

Adjourned to meet at 2 P. M.

October 28, 1886.

AFTERNOON SESSION.

Minutes of previous session read and approved.

Moved by Dr. C. H. James, that newly elected members be be introduced to the Society as they sign the Constitution. Carried.

The following persons having been recommended by the Board of Directors, were elected to membership:

J. S. McCarrell, W. T. Chambers, T. S. Seely, C. C. Scott, H. S. Ainsworth.

On motion, the following persons were elected to honorary membership:

E. L. Talbot, Chicago; Geo. L. Field, Detroit; Geo. Elliott.

Dr. Talbot then made some remarks upon seventh subject. Subject further discussed by Dr. Smith and others.

After offering vote of thanks to visiting members, Dr. Harroun, Callahan, and Robinson, the Society adjourned to meet in Springfield, Ohio, October, 1887.

